# Hyperpolarized Carbon-13 Metabolic Imaging Differentiates Distinctive Molecular Phenotypes in Diffuse Midline Gliomas

**DOI:** 10.3390/molecules30214175

**Published:** 2025-10-24

**Authors:** Ilwoo Park, Rintaro Hashizume, Joanna Phillips

**Affiliations:** 1 Department of Radiology, Chonnam National University Medical School and Hospital, Gwangju 61469, Republic of Korea; 2 Department of Artificial Intelligence Convergence, Chonnam National University, Gwangju 61186, Republic of Korea; 3 Department of Data Science, Chonnam National University, Gwangju 61186, Republic of Korea; 4 Department of Radiology and Biomedical Imaging, University of California, San Francisco, CA 94143, USA; 5 Department of Pediatrics, University of Alabama at Birmingham, Birmingham, AL 35233, USA; 6 Department of Neurological Surgery and Pathology, University of California, San Francisco, CA 94143, USA

**Keywords:** diffuse midline glioma, pediatric brain tumor, hyperpolarized carbon-13, metabolic imaging, magnetic resonance imaging

## Abstract

Despite a specific histone mutation defining the unique genetic makeup, diffuse midline gliomas are heterogeneous tumors with a wide range of morphologic and molecular spectrum. We investigated the feasibility of using hyperpolarized carbon-13(^13^C) MR metabolic imaging to differentiate distinctive molecular features from two H3K27M-mutant, biopsy-originated diffuse midline glioma xenografts. ^13^C MR metabolic imaging data were acquired on a 3T scanner from 12 rats that had been implanted with SF8628 or SF7761 diffuse midline glioma cells in brainstem, following injection of hyperpolarized [1-^13^C]pyruvate. Despite the two tumors’ similar appearance of T_2_-hyperintensity throughout the cerebellum and pons without contrast enhancement, ^13^C metabolic imaging data revealed that SF8627 had significantly higher ratios of lactate to pyruvate, lactate to total carbon, and normalized lactate than SF7761. Elevated lactate levels in SF8628 were associated with large amounts of lactate dehydrogenase (LDH)-A and carbonic anhydrase-IX staining in SF8628 compared to SF7761, which implied that the highly hypoxic condition in SF8628 appeared to contribute to the high level of LDH-A enzyme activity, which, in turn, induced the large conversion from hyperpolarized pyruvate to lactate. Our findings suggest that this advanced metabolic imaging technique may be used for the noninvasive characterization of molecular hypoxia and lactate dehydrogenase-A activity in these pediatric brainstem gliomas.

## 1. Introduction

Diffuse midline glioma is a new entity added to the 2016 World Health Organization Classification of Tumors of the Central Nervous System (CNS) [1]. It is a diffusely infiltrating astrocytic tumor, which generally develops in the pons, thalamus, and other midline CNS structures of children and young adults. Diffuse midline gliomas have a specific mutation at K27 position of histone H3 [2,3,4] and present aggressive clinical symptoms and dismal prognosis with a median survival time of 9 months [5,6,7]. Due to its diffusive nature and the intricacy of the brainstem location, surgical resection is impossible and biopsies have rarely been performed.

Several advances have been achieved in recent diffuse midline glioma research. Cell cultures derived from postmortem tumor tissue and patient biopsy have been established and orthotopic xenograft models created [8,9]. A combination of autopsy and biopsy samples have shed insight on the genetic and morphologic makeup of diffuse midline gliomas [3,10]. Despite the H3K27M mutation defining the unique genetic makeup of these diseases, diffuse midline gliomas are a heterogeneous group of tumors that display a wide range of characteristics on the morphologic and molecular spectrum [4,11,12]. Despite this heterogeneity, H3K27M-mutant diffuse midline gliomas are considered WHO grade IV tumors based on their dismal prognosis irrespective of their histologic grade. Histologic grading of H3K27M-mutant glioma, based on an assessment of features associated with aggressive disease, including elevated proliferation, necrosis, and microvascular proliferation, located in the thalamus suggest additional tumor features may retain prognostic value in specific anatomic regions [13].

Magnetic resonance imaging (MRI) is the standard imaging modality for diagnosis, monitoring of disease status, and treatment response in diffuse midline glioma [14]. Although conventional MRI techniques offer useful information about anatomic features, imaging features from MRI are heterogeneous with variable levels of necrosis, patterns of enhancement and edema [15]. In addition, the results obtained are not prognostic and are incapable of assessing tumor molecular characteristics and tissue function associated with effective therapy [16]. While positron emission tomography has been extensively used for evaluating tumor metabolism in oncology clinics, its use in children with diffuse midline glioma is limited due to ionizing radiation and has produced non-specific and non-prognostic outcome [17,18,19]. The development of noninvasive metabolic imaging methods that can be used to render insight into the molecular features of diffuse midline glioma will be beneficial in identifying tumor molecular characteristics as well as tailoring appropriate treatment options based on molecular features. 

Dynamic Nuclear Polarization and the development of a dissolution process which retains polarization into the liquid state enable the real-time investigation of in vivo carbon-13 (^13^C) metabolism with more than 10,000-fold signal increase over conventional ^13^C methods [20]. Previous studies have demonstrated the promise of this technique for examining in vivo tumor metabolism for application in the management of cancer [21,22,23]. Hyperpolarized ^13^C pyruvate, labeled at the C1 position, has been most widely used as a substrate for evaluating metabolism in combination with high-resolution ^13^C magnetic resonance spectroscopic imaging (MRSI) or MRI, which can be integrated with anatomic MRI. Recent studies using the hyperpolarized substrate [1-^13^C]pyruvate have demonstrated the promise of this technique for examining in vivo tumor metabolism in brain tumors, including the differentiation of the tumor from normal brain tissue as well as the detection of early response to treatment in animal models of high-grade glioma [24,25,26,27]. Recent human studies in neuro-oncology and other areas using hyperpolarized ^13^C MR metabolic imaging showed the safety and feasibility of this technology for evaluating real-time metabolism in humans [28,29,30,31].

Here, we investigate the feasibility of using ^13^C MR metabolic imaging with hyperpolarized [1-^13^C]pyruvate to characterize the molecular features of two H3K27M-mutant, biopsy-originated diffuse midline glioma. ^13^C MRSI data were acquired on a 3T scanner from 12 rats that had been implanted with SF8628 or SF7761 diffuse midline glioma cells in the brainstem. The metabolic imaging data were compared with histopathologic features from immunohistochemical analysis to determine whether specific metabolic features obtained from hyperpolarized ^13^C MR metabolic imaging can be used to differentiate distinct molecular phenotypes.

## 2. Results

**Figure 1:**
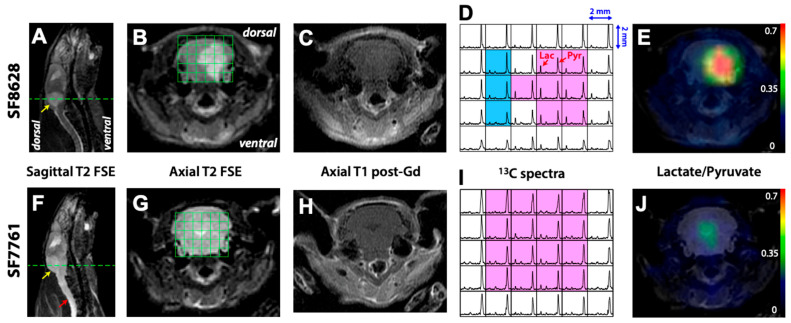
Examples of hyperpolarized ^13^C MR metabolic imaging data from two rats bearing SF8628 (**A**–**E**) or SF7761 (**F**–**J**) diffuse midline gliomas showing sagittal T_2_-weighted images (**A**,**F**), axial T_2_-wegihted images in brainstem (**B**,**G**), axial T_1_ post-gadolinium images in brainstem (**C**,**H**), ^13^C spectra (**D**,**I**) from the green spectral arrays in B and G, and the corresponding map of lactate-to-pyruvate ratio overlaid on top of the axial T_2_-weighted images (**E**,**J**). The yellow arrows in (**A**,**F**) indicate T_2_-hyperintensity, and the horizontal dashed lines delimit the location of 5.4 mm axial slice of the ^13^C spectra shown in (**D**,**I**). The red arrow indicates T_2_ abnormality that spread throughout the entire brainstem in the SF7761 bearing rat. The pink and blue boxes in (**D**,**I**) represent voxels over the T_2_-hyperintense lesion and contralateral normal brain tissue, respectively. The ^13^C spectra and the map of lactate-to-pyruvate show highly elevated lactate signal in SF8628 tumor compared to both contralateral brain and SF7761 tumor.

Figure 1 shows representative data from rats injected with SF8628 and SF7761 diffuse midline glioma cells. Figure 1A,B,F,G represent T_2_-weighted sagittal and axial images for SF8628 and SF7761 tumors, respectively. The sagittal and axial T_2_ fast spin echo (FSE) images showed regions of T_2_-hyperintensity expanding throughout the pons and cerebellum for both tumors (yellow arrow in Figure 1A,F). SF8628 had a relatively well-defined margin of T_2_ lesion (Figure 1A,B). In contrast, SF7761 showed T_2_ abnormality, which spread throughout the entire brainstem (Figure 1F,G) and extended to the spinal cord (red arrow in Figure 1F). There was no contrast enhancement on T_1_ post-gadolinium (Gd) images for both tumors (Figure 1C,H).

The axial T_2_-weighted images in Figure 1B,G show a ^13^C MRSI grid over the brainstem for SF8628 and SF7761 tumors, respectively. The horizontal dotted lines in Figure 1A,F delimit the location of 5.4 mm axial slice of ^13^C MRSI data presented in Figure 1D,I. The corresponding ^13^C spectra in Figure 1D,I demonstrated the spatial distribution of ^13^C-labeled pyruvate and its metabolic product lactate with high signal intensity in the entire brainstem region. SF8628 produced elevated lactate signal in the T_2_ lesions (pink voxels in Figure 1D) and relatively small lactate peaks in the contralateral normal brain (blue voxels in Figure 1D). The corresponding region of T_2_-hyperintensity exhibited a high ratio of lactate to pyruvate (Lac/Pyr, Figure 1E). In contrast, SF7761 did not show elevated levels of lactate in the T_2_ lesions (pink voxels in Figure 1I) and exhibited relatively low level of Lac/Pyr (Figure 1J).

**Figure 2:**
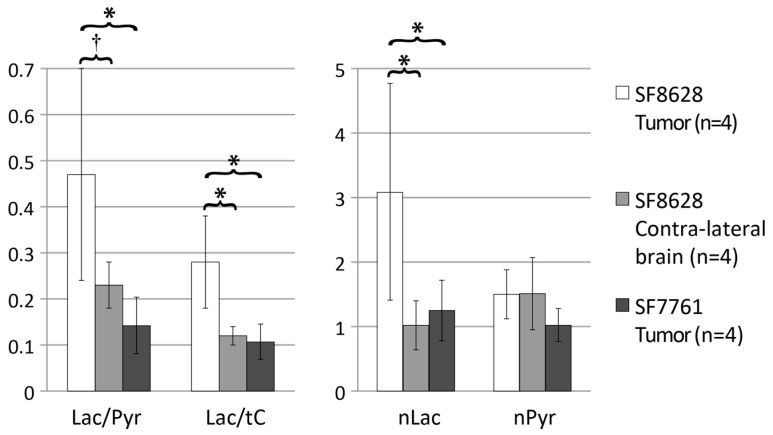
Comparison of ^13^C metabolite parameters between SF8628 tumor, SF8628 contralateral brain and SF7761 tumor. SF8628 tumor exhibited significantly higher levels of the ratio of lactate to pyruvate (Lac/Pyr), lactate to total carbon (Lac/tC) and normalized lactate (nLac) than contralateral brain tissue and SF7761 tumor. The levels of normalized pyruvate (nPyr) were similar between three tissues. * Significant difference with *p* < 0.01. ^†^ Significant difference with *p* < 0.03.

The summary of ^13^C parameters from the hyperpolarized ^13^C MR metabolic imaging data between two diffuse midline gliomas and the contralateral normal brain is shown in Table 1 and Figure 2. The mean Lac/Pyr and the ratio of lactate to total carbon (Lac/tC, tC: a sum of lactate, pyruvate-hydrate, alanine and pyruvate) and normalized lactate (nLac) in T_2_-hyperintensity from rats bearing SF8628 diffuse midline glioma were significantly higher than those in T_2_-hyperintensity from rats bearing SF7761 diffuse midline glioma (*p* < 0.01 for Lac/Pyr and Lac/tC, *p* < 0.03 for nLac). In addition, the levels of Lac/Pyr, Lac/tC and nLac in SF8628 tumor were significantly higher than those in contralateral brain tissue (*p* < 0.01). The levels of Lac/Pyr, Lac/tC and nLac in SF7761 tumors were similar to those of contralateral normal brain tissue measured from rats with SF8628 tumor (*p* > 0.05). The levels of normalized pyruvate (nPyr) were similar between SF8628 tumor, SF7761 tumor and contralateral brain tissue (*p* > 0.5 in all three pairwise comparison).

**Figure 3:**
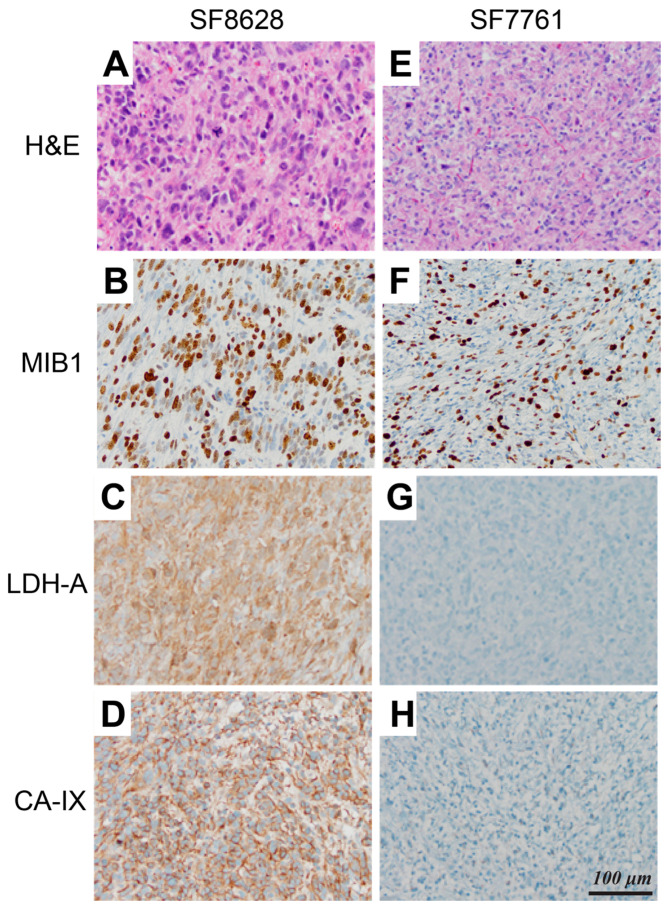
Comparison of histologic and immunohistologic features of SF8628 and SF7761 diffuse midline gliomas. (**A**,**B**,**E**,**F**) Both tumors displayed infiltrative, highly proliferative tumors recapitulating the histopathology of pediatric diffuse midline glioma with slightly higher proliferation in SF8628, as indicated by MIB1. (**C**,**D**,**G**,**H**) SF8628 tumors displayed increased expression of LDH-A and CA-IX compared to SF7761. The scale bar in F applies to all sub-figures (**A**) through (**H**). H&E, hematoxylin and eosin; MIB1, Molecular Immunology Borstel1; LDH-A, lactate dehydrogenase-A, CA-IX, carbonic anhydrase-IX.

Figure 3 shows histologic and immunohistologic comparisons between SF8628 and SF7761 xenografts. Both diffuse midline glioma models displayed infiltrative, highly proliferative tumors that recapitulated the histopathology of pediatric diffuse midline glioma (Figure 3A,B,E,F). SF8628, however, had more aggressive features than SF7761 with numerous atypical mitoses and prominent nuclear pleomorphism (Figure 3A,E). These histopathologic differences in the xenograft tumors mirrored differences in the human tumors from which they were derived as SF8628 had a histologic grade of IV while SF7761 was histologically a grade II with the lack of necrosis or microvascular proliferation.

**Figure 4:**
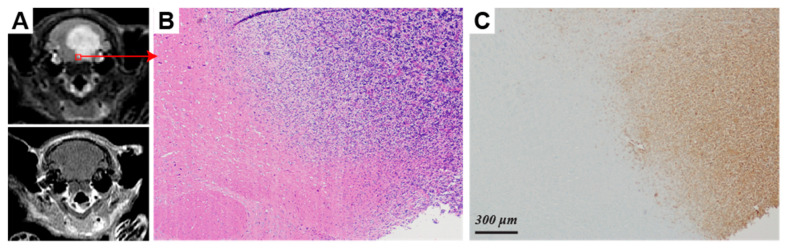
Pathologic features of a diffuse midline glioma (SF8628) and the corresponding radiographic feature. (**A**) A T_2_-weighted (**top**) and a T_1_ post- gadolinium (**bottom**) axial images demonstrate a non-enhancing, T_2_-hyperintense mass expanding the pons with infiltration into the adjacent cerebellum. (**B**) H&E stained section shows a highly cellular glioma with diffuse invasion into adjacent brain. (**C**) Immunostaining demonstrates LDH-A expression within the tumor cells. The scale bar in C applies to Figure 4B as well. The red square represents the region that corresponds to the pathologic images in (**B**,**C**).

Immunohistochemical analysis revealed a clear distinction in the molecular characteristics of two diffuse midline gliomas. In comparison to SF7761, SF8628 tumors had diffuse positive immunostaining for lactate dehydrogenase (LDH)-A (Figure 3C,G), an enzyme that catalyzes pyruvate-to-lactate conversion. SF8628 were also strongly positive for carbonic anhydrase (CA)-IX (Figure 3D), a downstream target of hypoxia-inducible factor-1 alpha (HIF-1α), compared to SF7761 (Figure 3H). In SF8628, the regions with elevated LDH-A immunostaining and marked infiltration of neoplastic astrocytes in the pons (Figure 4B,C) corresponded with T_2_-hyperintense lesions on T_2_-weighted FSE images (Figure 4A).

## 3. Discussion

This study demonstrates the feasibility of assessing heterogeneous metabolic profiles, associated with distinct molecular characteristics in human diffuse midline glioma orthotopic xenografts, using hyperpolarized ^13^C metabolic imaging with [1-^13^C]pyruvate as a substrate. Compressed sensing hyperpolarized 3D ^13^C MRSI data provided adequate signal to detect hyperpolarized [1-^13^C]pyruvate and its metabolic product lactate in brainstem. High spatial resolution of ^13^C spectra (0.022 cm^3^) enabled the comparison of ^13^C metabolic parameters between two diffuse midline gliomas and contralateral normal brain tissue.

All three of the ^13^C lactate parameters that were evaluated displayed significant differences between SF8628 and SF7761 tumors (Figure 2). SF8628 produced significantly higher levels of Lac/Pyr, Lac/tC and nLac than SF7761 (Table 1). Consistent with these imaging differences, immunohistochemical analysis of the two tumors revealed substantial differences in the levels of LDH-A and CA-IX (Figure 3). The robust expression of LDH-A and CA-IX in SF8628 corresponded with the elevated levels of lactate detected from the hyperpolarized ^13^C imaging. Similarly, the low level of lactate observed in the hyperpolarized ^13^C imaging of SF7761 xenografts was associated with the minimal expression of LDH-A and CA-IX.

The association between the hyperpolarized ^13^C lactate signal and the expression of LDH-A and CA-IX can be explained by a molecular response to hypoxia in cancer. Metabolically active tumors can become hypoxic, which drives the expression of hypoxia-inducible factors such as HIF-1α [32]. *LDHA*, a known transcriptional target of HIF-1A [33], encodes the enzyme LDH-A that catalyzes the inter-conversion of pyruvate and lactate [34]. CA-IX is also an indicator of cellular hypoxia [35,36]. Thus, a hypoxic metabolic phenotype of SF8628 likely contributed to the high level of LDH-A enzyme activity and the large conversion of ^13^C-label from hyperpolarized pyruvate to lactate. In contrast, less hypoxic SF7761 contributed to the low level of LDH-A enzyme activity, and as a result, relatively low lactate levels were produced from hyperpolarized ^13^C pyruvate. These results suggest that this advanced metabolic imaging technique may be used for the noninvasive characterization of molecular hypoxia and LDH-A activity in these pediatric brainstem gliomas.

Interestingly, the levels of normalized pyruvate were similar between T_2_-hyperintense lesions of SF8628 (1.0 ± 0.3), SF7761 (1.0 ± 0.3) and contralateral brain tissue from rats bearing SF8628 (1.1 ± 0.4), highlighting that the amount of pyruvate delivered via i.v. injection and taken up by these tissues were comparable between these different tissues. Given that these tumors are non-enhancing and different levels of lactate were observed in these three tissues, we can conclude that the majority of ^13^C lactate observed in our experiments came from the conversion of ^13^C pyruvate by tumor cells with minimal contributions from the vasculature.

Although MRI has been mainly used for the diagnosis and assessment of therapy response in diffuse midline gliomas, radiographic appearance obtained from the conventional MRI methods exhibits diverse imaging features [15]. Similarly, morphologic features obtained from MRI cannot provide the prediction of response to treatment in diffuse midline gliomas. Two diffuse midline glioma xenografts used in this study presented distinctive metabolic profiles based on hyperpolarized ^13^C MR imaging which reflect their distinct molecular phenotype. In contrast, their radiographic characteristics were comparable with high T_2_-hyperintensity, accompanied by the absence of contrast enhancement. Our findings suggest that metabolic information acquired from hyperpolarized ^13^C MR imaging can be used to differentiate two diffuse midline gliomas that have inherent differences in their molecular and histopathological makeups.

We believe that another potential application of this technique may be monitoring and evaluating response to treatment in patients with diffuse midline glioma. Cells undergoing growth arrest or apoptosis convert less lactate from hyperpolarized pyruvate [22,37]. Thus, the administration of hyperpolarized [1-^13^C]pyruvate to tumor-bearing subjects, followed by the real-time monitoring of conversion to [1-^13^C]lactate has allowed for not only the localization of malignant tissue, but also the study of therapy response. The reduced conversion of hyperpolarized pyruvate-to-lactate was shown to be linked to temozolomide-induced DNA damage and radiation-induced response in the rodent models of high-grade adult brain tumors [26].

The first human study using hyperpolarized ^13^C MR metabolic imaging was accomplished in prostate cancer patients and demonstrated the safety and feasibility of this technology for use in a clinical setting [30]. This has triggered great interest in applying this novel imaging method for volunteer cardiac studies [38] and adult patients with brain tumors [31]. Conceivable challenges in translating this imaging method to the clinic for children with diffuse midline glioma include the necessity of anesthesia for younger-age patients and ensuring adequate SNR in the brainstem, where the surrounding tissue is less perfused and far removed from RF receiver coil elements. This technical challenge is an area of rapid development and expected to be overcome in the near future. A recent pilot study demonstrated the safety and tolerability of hyperpolarized ^13^C MR metabolic imaging in pediatric patients with brain tumors [39].

While these early studies underscore the promise of hyperpolarized ^13^C MR imaging in a clinical setting, several practical considerations remain. The technique requires specialized hyperpolarization equipment and rapid dissolution systems that are not yet widely available, limiting accessibility to a few research centers with dedicated infrastructure. The short polarization lifetime and the need for precise timing between polarization, injection, and imaging acquisition further demand rigorous coordination and technical expertise. These logistical and cost-related constraints currently hinder broader implementation in both preclinical and clinical settings. From a translational perspective, the application of hyperpolarized ^13^C MRI in pediatric patients also raises considerations related to safety, feasibility, and standardization. Although [1-^13^C]pyruvate has demonstrated a favorable safety profile in adult clinical trials, additional validation is required to confirm its safety and optimal dosing in children, who may have distinct metabolic and physiological characteristics. Moreover, standardization of acquisition parameters, quantification methods, and data interpretation frameworks remains essential to ensure reproducibility across sites and studies. Establishing consensus protocols and quality control measures will be critical for integrating hyperpolarized ^13^C MRI into multicenter pediatric studies and, ultimately, for its translation into clinical practice as a robust metabolic imaging biomarker.

While we find the results from this study very promising, there are several limitations of this study. Diffuse midline gliomas are a very heterogeneous tumor. The primary pediatric human diffuse midline glioma cell lines that were used to generate intracranial tumors may not represent the whole spectrum of morphologic and metabolic characteristics of diffuse midline glioma. In addition, there may be differences in brain metabolism between humans and other species; therefore, the proper preparation of experimental setup as well as careful interpretation of the data are necessary to study human subjects. In addition, the relatively small sample size of animals included in this study constitutes a limitation that warrants consideration. The limited number of animals could affect the robustness of the conclusions. Furthermore, as the experiments were conducted under controlled laboratory conditions using xenografts with specific cell lines, the extent to which these findings can be generalized to other diffuse midline gliomas models or clinical contexts remains uncertain. Nonetheless, the consistent observation of the distinct imaging and molecular patterns supports the relevance of the results. Future investigations involving larger cohorts and complementary validation approaches will be valuable to strengthen and extend these conclusions.

## 4. Materials and Methods

### 4.1. Cell Culture and Implantation of Intracranial Tumors

Primary pediatric human diffuse midline glioma cell lines, SF8628 and SF7761, were obtained from the UCSF medical center in accordance with institutionally approved protocol. The diagnostic histopathology of the tissue obtained from the surgical biopsy was consistent with an infiltrative astrocytoma, WHO grade II for SF7761 and Grade IV for SF8628. Establishment of diffuse midline glioma cell cultures, from surgical specimens, and tumor cell modification for expression of firefly luciferase, for in vivo bioluminescence imaging, have been described [8]. Six-week-old male athymic rats (rnu/rnu, homozygous) purchased from Harlan (Indianapolis, IN, USA) were housed under aseptic conditions with filtered air and sterilized food, water, bedding, and cages. Tumor cells were implanted into the pontine tegmentum of athymic rats as previously described [40]. Briefly, rats were anesthetized by i.p. injection of a mixture containing ketamine (60 mg/kg) and xylazine (7.5 mg/kg) in 0.9% saline. A 1 cm sagittal incision was made along the scalp, and the skull suture lines were exposed. A small hole was created by puncture with a 25 g needle at 2.0 mm to the right of the midline and 9.6 mm behind the bregma. Using a sterile Hamilton syringe (Stoelting, Wood Dale, IL, USA), 1 × 10^6^ cells in 1 μL Hanks’ Balanced Salt Solution, without Ca^2+^ and Mg^2+^, were slowly injected into the pontine tegmentum at 9.6 mm deep from the inner base of the skull. All protocols were approved by the Institutional Animal Care and Use Committee.

### 4.2. Animal Preparation

A total of 12 male athymic rats with human diffuse midline glioma xenograft tumors (8 injected with SF8628 cell lines and 4 with SF7761 cell lines) were included in this study. The timing of imaging data acquisition was based on the animals’ clinical symptoms, including weight loss, abnormal posture, lethargy, decreased activity, ataxia, and head tilt. Due to the distinct growth characteristics of the two cell lines, rats implanted with the SF8628 cell line went through imaging experiments at 59 ± 7 days (mean ± SD), and those implanted with the SF7761 cell line at 103 ± 3 days (mean ± SD) after the tumor implantation. The median weight of the animals at the time of the imaging experiment was 280 g. The following procedures were carried out for each imaging experiment. The animal was anesthetized with approximately 1.5% isoflurane and placed on a warm pad. For the i.v. injection of hyperpolarized pyruvate solution, a catheter was placed into the tail vein. The animal was then brought to a warm pad located in the RF coil of the MR scanner. Anesthesia was continued with a constant delivery of approximately 1.5% isoflurane through a tube to a cone fixed over the rat’s nose and mouth while the rat underwent the imaging experiment. Rats’ body temperature was maintained using a warm pad placed inside the RF coil.

### 4.3. Polarization Procedure

For the polarization of pyruvate, 35 μL of [1-^13^C]pyruvate mixed with 15 mM OX063 trityl radical (GE Healthcare, Oslo, Norway) and 1.5 mM Gadolinium (Gd)-DOTA was polarized using a HyperSense polarizer (Oxford Instruments, Abingdon, UK) at 3.35 T and 1.4 K by irradiation with 94.1 GHz microwaves using the method described previously [20]. After approximately 60 min of microwave irradiation, the mixture was rapidly dissolved in a saline solution with 5.96 g/L Tris (40 mM), 4.00 g/L NaOH (100 mM), and 0.1 mg/L Na2 ethylenediaminetetraacetic acid (EDTA). The final dissolved solution had a concentration of 100 mM pyruvate and pH ~7.5. A sample from the dissolved pyruvate solution with a volume of 2.7 mL was injected into the tail vein of the rat over 10 s duration.

### 4.4. ^1^H and ^13^C MR Imaging

All imaging experiments were performed using a 3 T clinical MRI system (GE Healthcare, Waukesha, WI, USA) with a multinuclear spectroscopy (MNS) hardware package, and a custom-designed ^1^H/^13^C coil with a quadrature ^13^C channel and linear ^1^H channel with an 8 cm inner coil diameter and 9 cm length.

The following ^1^H and ^13^C data were acquired in sequence for each imaging session: (1) T_2_-weighted axial images using a FSE sequence (TE/TR = 60/4000 ms, 8 cm FOV, 256 × 192 matrix, 2 mm slice thickness and 4 NEX); (2) compressed sensing ^13^C 3D MRSI data (TE/TR = 140/215 ms, phase encoding in x and y axes, flyback echo-planar spectroscopic imaging readout in *z*-axis, 20 × 16 × 16 matrix, 2 × 2 × 5.4 mm spatial resolution) [41] acquired at 20 s from the start of the pyruvate injection [42]; and (3) T_1_-weighted axial images using a spin-echo sequence (TE/TR = 10/700 ms, 8 cm FOV, 320 × 192 matrix, 1.2 mm slice thickness, and 6 NEX) after the injection of 0.1 mL Gd-DTPA (approximately 0.2 mmol/kg).

### 4.5. Immunohistochemical Analysis

Following completion of the imaging experiments, animals were euthanized, and the brains were extracted and fixed in 4% formalin prepared in phosphate buffer. Tissue samples were subsequently dehydrated through a graded ethanol series and embedded in Paraplast Plus wax (McCormick Scientific, Berkeley, MO, USA). Sections of 5 µm thickness were prepared and stained with hematoxylin and eosin (H&E) as well as specific antibodies. The primary antibodies employed included Ki-67 (clone MIB-1; Ventana Medical Systems, Inc., Oro Valley, AZ, USA), LDH-A (Cell Signaling Technology, Danvers, MA, USA), and CA-IX (Novus Biologicals, Centennial, CO, USA), targeting cellular proliferation, LDH-A, and HIF-1 expression, respectively. Comparative histological and immunohistochemical evaluations of SF8628 and SF7761 tumors were performed on tissue regions approximately corresponding to the centers of the spectroscopic voxels. Immunostaining was carried out on a Benchmark XT automated stainer (Ventana Medical Systems, Inc.) using the iView detection kit. Digital images were captured using an Olympus DP72 microscope camera.

### 4.6. Data Processing and Analysis

Processing of the ^13^C MRSI data followed previously established procedures [43]. In summary, raw readout signals were reorganized into a four-dimensional array, and only k-space data acquired during the constant gradient portions of the flyback trajectory were retained. The time-domain data were apodized using a 16 Hz Gaussian filter and zero-filled to 256 points. Spectral reconstruction was achieved via a four-dimensional Fourier transform, generating a three-dimensional spatial array of spectra. A linear phase correction was subsequently applied along the flyback dimension to compensate for k-space point offsets. Missing k-space information was reconstructed using an iterative compressed sensing algorithm based on the L1-norm. A detailed description of this reconstruction approach has been reported elsewhere [44].

For quantification of ^13^C metabolites, Lac/Pyr and Lac/tC were calculated. In addition, the lactate and pyruvate signals in the brainstem were normalized with respect to the relative signals in normal brain in the supratentorial region (Supplementary Figure S1). Regions of interests were manually contoured on T_2_ FSE images for the T_2_-hyperintense lesion and the percentage of T_2_ lesion volume was calculated for each voxel. In order to evaluate the spatial variation of ^13^C metabolites, the mean ^13^C parameters were compared between the T_2_-hyperintense lesion (voxels with >70% T_2_ lesion), and contralateral normal brain (voxels with 100% normal appearing brain tissue). The ^13^C parameters from the T_2_-hyperintense lesion of SF8628 and SF7761 xenografts and the contralateral normal brain of SF8628 xenografts were compared using a paired or Student’s *t*-test. All statistical analyses were performed using MATLAB (version 7.0; The MathWorks, Inc., Natick, MA, USA).

## 5. Conclusions

Hyperpolarized ^13^C metabolic imaging was able to detect differences in metabolism associated with distinct molecular characteristics in two human diffuse midline glioma orthotopic xenografts. The elevated lactate signal observed in the diffuse midline glioma xenografts was associated with increased levels of LDH-A and CA-IX. Thus, this novel metabolic imaging method may be used to noninvasively characterize molecular hypoxia and LDH-A activity in diffuse midline gliomas and may be useful to assist in monitoring disease progression and response to treatment response.

## Figures and Tables

**Table 1:** Summary of hyperpolarized ^13^C metabolite quantification. Metabolite values are mean ± SD.

	Lactate/Pyruvate ^1,2^	Lactate/Total Carbon ^1,2^	Normalized Lactate ^1,3^	Normalized Pyruvate
SF8628 tumor (n = 8)	0.70 ± 0.24	0.36 ± 0.08	2.9 ± 1.1	1.0 ± 0.3
SF8628 contralateral brain (n = 8)	0.28 ± 0.11	0.18 ± 0.07	1.1 ± 0.3	1.1 ± 0.4
SF7761 tumor (n = 4)	0.14 ± 0.06	0.11 ± 0.04	1.3 ± 0.5	1.0 ± 0.3

^1^ Significant difference between SF8628 tumor and SF8628 contralateral brain (*p* < 0.01, paired *t*-test). ^2^ Significant difference between SF8628 tumor and SF7761 tumor (*p* < 0.01, Student’s *t*-test). ^3^ Significant difference between SF8628 tumor and SF7761 tumor (*p* < 0.03, Student’s *t*-test).

## Data Availability

The datasets presented in this article are not readily available owing to ethical and institutional restrictions related to the use of animal experimental data. Requests to access the datasets should be directed to the corresponding author.

## Supplementary Materials

The following supporting information can be downloaded at: https://www.mdpi.com/article/10.3390/molecules30214175/s1, Figure S1: Graphical illustration of two brain regions used for the quantification of hyperpolarized 13C imaging data. In addition to calculating the ratio of lactate-to-pyruvate and lactate-to-total carbon in infratentorial brain (C), lactate and pyruvate signal were normalized by their respective values from normal appearing brain tissue in supratentorial region (A).
