# Supplementary material for: Hyperpolarized Carbon-13 Metabolic Imaging Differentiates Distinctive Molecular Phenotypes in Diffuse Midline Gliomas
Source: Molecules. 2025 Oct 24;30(21):4175. doi: 10.3390/molecules30214175 (PMC12608448; doi:10.3390/molecules30214175)
Supplement: Supplementary file 1 [file molecules-30-04175-s001.zip › molecules-3902208-supplementary.pdf]

Supplementary Materials

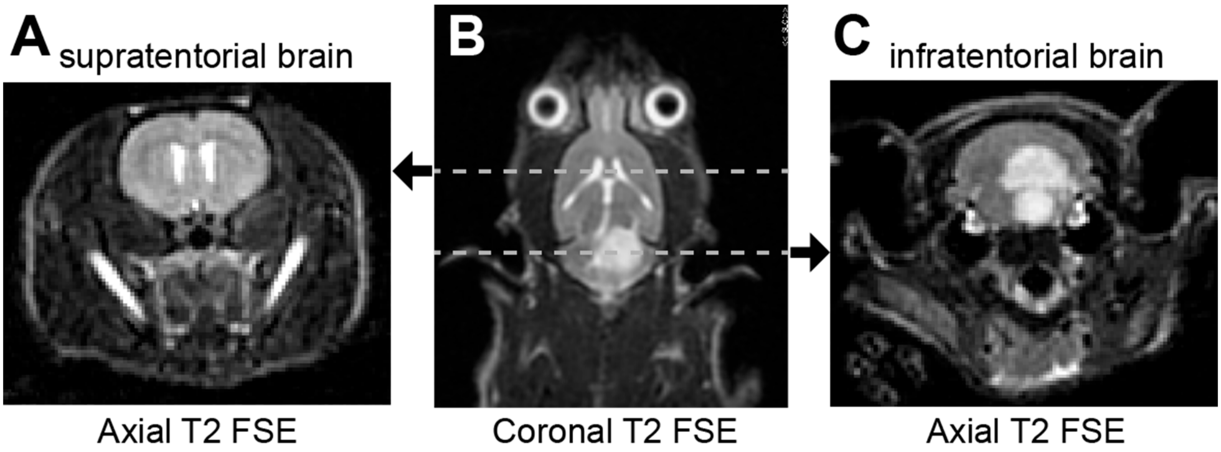

Supplementary Figure S1. Graphical illustration of two brain regions used for the quantification of hyperpolarized ^{13}C imaging data. In addition to calculating the ratio of lactate-to-pyruvate and lactate-to-total carbon in infratentorial brain (C), lactate and pyruvate signal were normalized by their respective values from normal appearing brain tissue in supratentorial region (A).
